# Accelerated variant curation from scientific literature using biomedical text mining

**DOI:** 10.17912/micropub.biology.000578

**Published:** 2022-06-01

**Authors:** Rishab Mallick, Valerio Arnaboldi, Paul Davis, Stavros Diamantakis, Magdalena Zarowiecki, Kevin Howe

**Affiliations:** 1 European Molecular Biology Laboratory, European Bioinformatics Institute, Wellcome Trust Genome Campus, Hinxton, Cambridge CB10 1SD, UK; 2 Division of Biology and Biological Engineering 140-18, California Institute of Technology, Pasadena, CA 91125, USA

## Abstract

Biological databases collect and standardize data through biocuration. Even though major model organism databases have adopted some automation of curation methods, a large portion of biocuration is still performed manually. To speed up the extraction of the genomic positions of variants, we have developed a hybrid approach that combines regular expressions, Named Entity Recognition based on BERT (Bidirectional Encoder Representations from Transformers) and bag-of-words to extract variant genomic locations from *C. elegans* papers for WormBase. Our model has a precision of 82.59% for the gene-mutation matches tested on extracted text from 100 papers, and even recovers some data not discovered during manual curation. Code at: https://github.com/WormBase/genomic-info-from-papers

**Figure 1. Overview of the data extraction process and key results:**
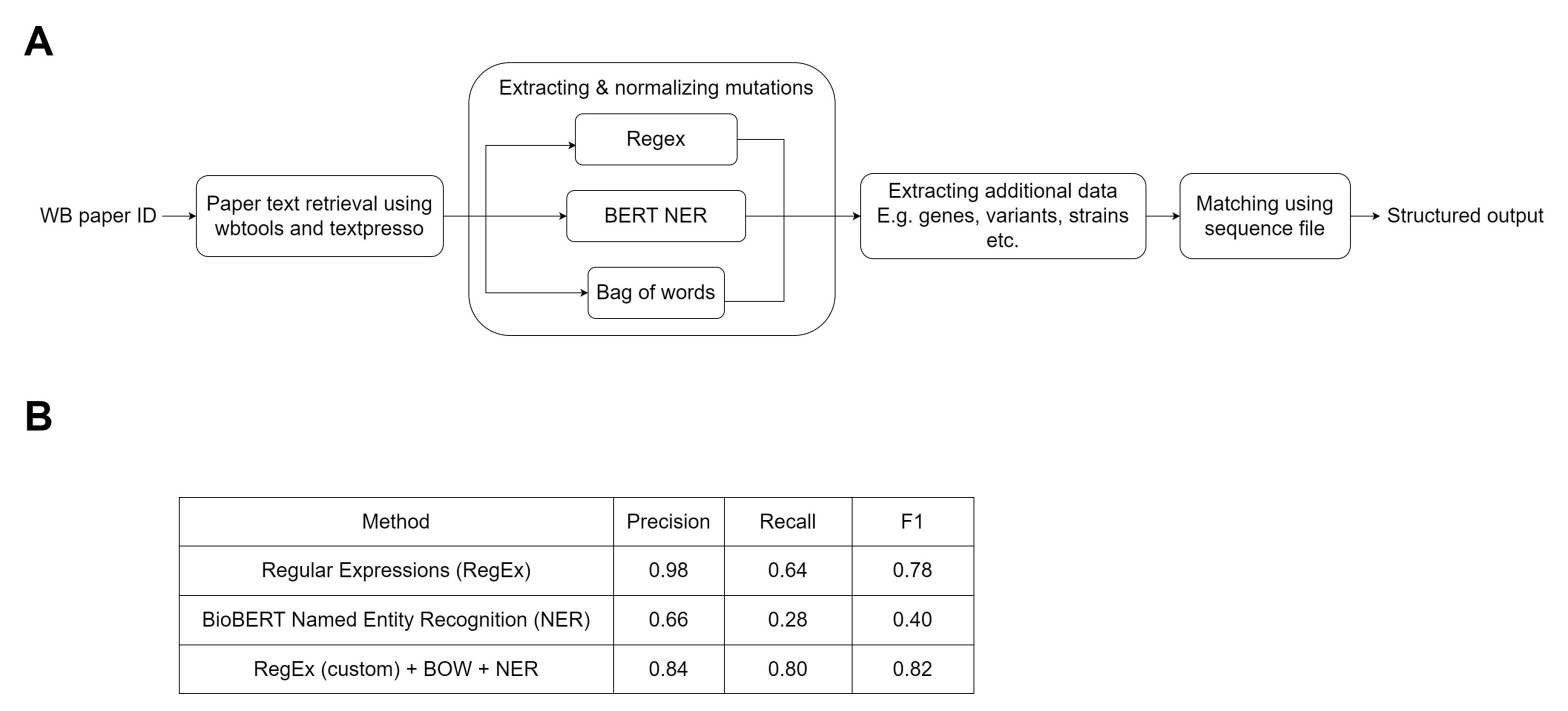
(A) Using database publication IDs as input, the sentences from the corresponding papers are extracted using wbtools or TextPresso, and then processed by the hybrid system where all mutation mentions are extracted. Additional information relevant for curation is then extracted using bag-of-words. The gene and mutation matches are verified by validating that the reference base/codon is possible in at least one transcript in the gene. The final matches and additional data (eg strain name) are then presented in a structured output for validation by curators before being used to update the database. (B) Metrics on sentence classification with mutation mention tested on IDP4+ corpus.

## Description

WormBase already uses entity recognition to scan newly published papers for the names of new strains and variants. However, the current method for adding genomic location details to variant records in WormBase involves the biocurators reading the publication to identify the relevant data and extract it. However, as more and more papers are published, due to technological improvements such as the adoption of CRISPR-Cas9, it becomes increasingly difficult to keep up with the number of new variants published every day. The variant genomic location data is also often not easily tractable, as the genomic location can be expressed in exact genomic location, eg. chr1:1004352, or relative location eg. Arg-107-Cys. Previous work in this field such as MutationFinder (Caporaso et al. 2007) and tmVar (Wei et al. 2013) are able to extract basic protein changing mutations (e.g. Q115P) from publications using Regular Expressions (RegEx). Nala uses Conditional Random Fields (CRF) for Named Entity Recognition (NER), and it is able to extract mutations in natural language form (e.g. ‘glutamic acid was substituted by valine at residue 6’) (Cejuela et al. 2017). Others explore the hybrid approach of merging annotations from RegEx, dictionary annotation and deep learning based on Bidirectional Long Short Term Memory Conditional Random Fields (BiLSTM-CRF) (Yepes et al. 2018). In this work, we are attempting to extract *C. elegans* protein-coding variants from recently published papers, and associate those with additional data, enough to be able to significantly speed up our variation curation process.

We first tested our hybrid model on the IDP4+ corpus, which has structured data, allowing us to calculate the performance on individual sentences. Figure 1B reports Precision, Recall, and F1 scores of the regex and NER blocks of our pipeline, as well as the overall statistics for the final hybrid solution. We then tested our pipeline on a set of 100 papers, which had been previously associated with sequenced variants by WormBase curators. We found that 93 contained some genomic location information, which we could extract. Some of this data would not be enough to extract all the data needed by the data model, but it can alert us to the presence of variation data in the paper. Data from these papers were previously manually curated and contained 2433 gene-mutation matches (which we consider the ground truth). Our automated pipeline was able to extract variant genomic data from 53 papers and find a total of 2314 mutation mentions (1694 from NER and 620 from RegEx). However, due to known limitations in the current iteration, only the mutations from the RegEx block were normalized, i.e. 73.2% of the total mutation mentions from NER were ignored, for example because they were indels, or contained incomplete data. Afterwards, using the protein sequence file in FASTA format, 977 gene-mutation matches were validated. During cross-checking these matches and manually examining the apparent false positives, some new matches were discovered that were previously missed during manual curation and added to the ground truth. After this update, the precision rate was 82.59% (TP: 807, FP: 170). This result is in line with the results found on the IDP4+ corpus (Cejuela et al. 2017). We also manually checked the 47 papers for which we could not extract variants, and we found in all cases this was due to the fact that the information we had curated was embedded in figures or tables, which we had not been able to extract the text from.

Extracting and curating new variants manually from the scientific literature is very time-consuming, and having an automated system drastically reduces the curation turnaround. For new papers this approach allows for the quick triage of a large number of hits to identify targets for curation. Moreover, the pipeline can be easily applied to older papers to find data previously missed. Certain areas will remain difficult - papers where it is uncertain which genome or annotation version was used, or protein-coding changes incorrectly annotated by authors. But - as we have demonstrated - an automated pipeline is able to extract data and flag it up to curators with high accuracy - allowing curators to focus their attention on slightly tricker variants or papers where there are known targets. Further work could be done to improve the mutation normalization block, as in the current iteration most of the output from the NER block (potentially making up more than 70% of the total mutations to be identified) is currently discarded as the normalization is comparatively harder than the output from the RegEx block, which is already close to the required format. However, if a mutation is an important focus of the paper, more often than not the same mutation will be discussed several times in the paper, and if only one of those mentions contains enough data, the genomic location could be accurately curated. Increasing the size of the training set, using active learning as more papers are processed, and verifying the model predictions by curators would further improve performance. It has been previously observed that a BiLSTM-CRF performs comparatively or in certain cases even better than a BERT based NER (Gao et al. 2021) , thus our NER+RegEx solution seems suitable for good performance on the semi-structured data of protein changing mutations. Further work can also include better parsing of text from tables and figures and further optimization of the normalisation and validation code. However, this proof-of-principle has shown that automatic extraction of protein-coding variants from primary scientific literature can dramatically improve the speed and ease by which we can curate variants.

## Methods

We have based our model on the one proposed earlier (Yepes et al. 2018), but we have further customized the hybrid pipeline (Figure 1A) to better suit the kind of data we are interested in extracting. The regular expression block consists of RegEx developed by tmVar and MutationFinder (Caporaso et al. 2007; Wei et al. 2013) downloaded from https://www.ncbi.nlm.nih.gov/research/bionlp/Tools/tmvar/ and http://mutationfinder.sourceforge.net/ both unversioned, which were then modified to capture a wider set of mutations and a few additional custom RegEx, which included an additional dictionary of words (bag-of-words) developed after studying curator remarks at WormBase (available at https://github.com/WormBase/genomic-info-from-papers). To extract text snippets that contain genomic location information, we used BioBERT “base cased” v1.1 (Lee et al. 2020), which uses English Wikipedia, BooksCorpus, PubMed abstracts, and PubMed Central (PMC) full-text articles as the corpus was applied as pre-trained weights (available in HuggingFace model hub https://huggingface.co/dmis-lab/biobert-base-cased-v1.1 , updated Oct 14, 2020) and fine-tuned on IDP4+, a large corpus of genetic and protein mutation mentions (Cejuela et al. 2017). The RegEx block was fairly successful at capturing most of the basic mutations. For this reason, we focused on building a meaningful training set for the BioBERT block, and the training set was further filtered to contain only mutations in natural language form, such as “glycine to arginine substitution at codon 20” and “deletion of 10 and 8 residues from the N- and C-terminals”. An 80/20 splitting of the IDP4+ corpus was done to create training and test sets.

The full workflow is as follows. First, the user inserts the WormBase ID of the paper to be processed. The paper text is pulled using TextPresso https://textpressocentral.org accessed June/July 2021 (Muller et al. 2018) or - when not available in TextPresso - converted on the fly from PDFs using the WormBase text mining library wbtools ( https://github.com/WormBase/wbtools , retrieved June 2021). Each sentence from the paper text is processed by the model: first, the sentence is processed by the RegEx block. If no basic mutations are present in the sentence, it is processed by the NER block. The mutations are then normalized to the same format, e.g. V600E, for downstream processing.

Then, additional relevant data like genes, strains, variation type (e.g. deletion, insertion), generation method (e.g. EMS, ENU, CRISPR-Cas9), and others are extracted, using specific dictionaries created from the current WormBase dataset and data models. The pipeline investigates if the reference base is possible in that protein, e.g. for MEC-5 G109E, we check that at least one transcript has the nucleotide sequence GGN in position 109, using WormBase genome and annotation files, and only verified mutations are progressed. The final structured output presented to the curators for validation contains these gene mutation matches, the extra data available (strain, gene, allele name), and the complete sentences the matches were found in. Further metadata which we have found useful includes; the number of times the mutation was mentioned in the paper, the number of genes the mutation could be matched to, and other very similar mutations (e.g. G218D and G218L). These metadata act as additional quality control during subsequent curation, it would for example be quite unlikely that the same mutation G359A would be found in two different genes in the same paper, so the curator can be alerted to the fact it is possible in more than one gene in the paper, and be the arbitrator of which is the correct association.
